# Supplementary material for: The Skeletal Effects of Tanshinones: A Review
Source: Molecules. 2021 Apr 16;26(8):2319. doi: 10.3390/molecules26082319 (PMC8073409; doi:10.3390/molecules26082319)
Supplement: Supplementary file 1 [file molecules-26-02319-s001.zip › molecules-1138399-supplementary.pdf]

molecules

Supplementary Material

The Skeletal Effects of Tanshinones: A Review

Sophia Ogechi Ekeuku, Kok-Lun Pang and Kok-Yong Chin *

Department of Pharmacology, Faculty of Medicine, Universiti Kebangsaan Malaysia, Level 17, Preclinical Building, Jalan Yaacob Latif, Bandar Tun Razak, 56000 Cheras Kuala Lumpur, Malaysia. virgosapphire2088@yahoo.com (S.O.E.); pangkoklun@ukm.edu.my (K.-L.P.)

* Correspondence: chinkokyong@ppukm.ukm.edu.my; Tel.: +60-3-91459573

Table S1. PRISMA Checklist.

Section/Topic	#	Checklist Item	Reported on Page #
		**TITLE**	
Title	1	Identify the report as a systematic review, meta-analysis, or both.	1
		**ABSTRACT**	
Structured summary	2	Provide a structured summary including, as applicable: background; objectives; data sources; study eligibility criteria, participants, and interventions; study appraisal and synthesis methods; results; limitations; conclusions and implications of key findings; systematic review registration number.	1
		**INTRODUCTION**	
Rationale	3	Describe the rationale for the review in the context of what is already known.	2
Objectives	4	Provide an explicit statement of questions being addressed with reference to participants, interventions, comparisons, outcomes, and study design (PICOS).	2
		**METHODS**	
Protocol and registration	5	Indicate if a review protocol exists, if and where it can be accessed (e.g., Web address), and, if available, provide registration information including registration number.	-
Eligibility criteria	6	Specify study characteristics (e.g., PICOS, length of follow-up) and report characteristics (e.g., years considered, language, publication status) used as criteria for eligibility, giving rationale.	2
Information sources	7	Describe all information sources (e.g., databases with dates of coverage, contact with study authors to identify additional studies) in the search and date last searched.	2
Search	8	Present full electronic search strategy for at least one database, including any limits used, such that it could be repeated.	3
Study selection	9	State the process for selecting studies (i.e., screening, eligibility, included in systematic review, and, if applicable, included in the meta-analysis).	3
Data collection process	10	Describe method of data extraction from reports (e.g., piloted forms, independently, in duplicate) and any processes for obtaining and confirming data from investigators.	3
Data items	11	List and define all variables for which data were sought (e.g., PICOS, funding sources) and any assumptions and simplifications made.	3

Risk of bias in individual studies	12	Describe methods used for assessing risk of bias of individual studies (including specification of whether this was done at the study or outcome level), and how this information is to be used in any data synthesis.	-
Summary measures	13	State the principal summary measures (e.g., risk ratio, difference in means).	4–8
Synthesis of results	14	Describe the methods of handling data and combining results of studies, if done, including measures of consistency (e.g., I^2) for each meta-analysis.	-

Table S2. Literature Search Results.

Author	Year	Title	Journal	Type
Hong-Hee Kim, Jung Ha Kim, Han Bok Kwak, Hao Huang, Song-Hee Han, Hyunil Ha, Soo Woong Lee, Eun-Ran Woo, Zang Hee Lee	2004	Inhibition of osteoclast differentiation and bone resorption by tanshinone IIA isolated from *Salvia miltiorrhiza* Bunge.	Biochemical pharmacology	Research
Liao Cui, Tie Wu, Yu-yu Liu, Yi-feng Deng, Chun-mei Ai, Huai-qing Chen	2004	Tanshinone prevents cancellous bone loss induced by ovariectomy in rats.	Acta pharmacologica Sinica	Research
Song-Yi Lee, Doo-Youn Choi, Eun-Rhan Woo	2005	Inhibition of osteoclast differentiation by tanshinones from the root of *Salvia miltiorrhiza* Bunge	Archives of Pharmacal Research	Research
Han Bok Kwak, Daum Yang, Hyunil Ha, Jong Ho Lee, Ha Neui Kim, Eun Ran Woo, Seungbok Lee, Hong Hee Kim, Zang Hee Lee	2006	Tanshinone IIA inhibits osteoclast differentiation through down-regulation of c-Fos and NFATc1.	Experimental & molecular medicine	Research
Han Bok Kwak, Hyun-Min Sun, Hyunil Ha, Ha-Neui Kim, Jong-Ho Lee, Hong-Hee Kim, Hong-In Shin, Zang Hee Lee	2008	Tanshinone IIA suppresses inflammatory bone loss by inhibiting the synthesis of prostaglandin E2 in osteoblasts.	European journal of pharmacology	Research
Hae-Kyung Kim, Eun-Rhan Woo, Hae-Won Lee, Hyung-Rae Park, Hyun-Nam Kim, Yeon-Kwan Jung, Je-Yong Choi, Soo-Wan Chae, Hyung-Ryong Kim, Han-Jung Chae	2008	The Correlation of Salvia miltiorrhiza Extract–Induced Regulation of Osteoclastogenesis with the Amount of Components Tanshinone I, Tanshinone IIA, Cryptotanshinone, and Dihydrotanshinone	Immunopharmacology and Immunotoxicology	Research
Hye Joo Kim, Seong Hwan Kim	2010	Tanshinone IIA enhances BMP-2-stimulated commitment of C2C12 cells into osteoblasts via p38 activation.	Amino acids	Research
Yanmeng Zhou, Yubo Liu, Yunsheng Gao	2010	Effect of tanshitone on prevention and treatment of retinoic acid induced osteoporosis in mice	China journal of Chinese materia medica	Research
Shiying ZHANG, Jiguang LIU, Gang ZHAO	2014	Tanshinone type IIA inhibits osteoprotegerin and osteoclast differentiation factor expression at relapse stage after orthodontic tooth movement	Chinese Journal of Tissue Engineering Research	Research

Kejun Qian, Huazhong Xu, Teng Dai, Keqing Shi	2015	Effects of Tanshinone IIA on osteogenic differentiation of mouse bone marrow mesenchymal stem cells	Naunyn-Schmiedeberg's Archives of Pharmacology	Research
Jia Li, Chongru He, Wenwen Tong, Yuming Zou, Dahe Li, Chen Zhang, Weidong Xu	2015	Tanshinone IIA blocks dexamethasone-induced apoptosis in osteoblasts through inhibiting Nox4-derived ROS production.	International journal of clinical and experimental pathology	Research
Preety Panwar, Liming Xue, Kent Søe, Kamini Srivastava, Simon Law, Jean-Marie Delaisse, Dieter Brömme	2017	Journal of bone and mineral research : the official journal of the American Society for Bone and Mineral Research	An Ectosteric Inhibitor of Cathepsin K Inhibits Bone Resorption in Ovariectomized Mice.	Research
Jun Yao, Shiting Ma, Wenyu Feng, Yan Wei, Huiping Lu, Gang Zhong, Zhengyuan Wu, Hongtao Wang, Wei Su, Jia Li	2018	Tanshinone IIA protects against polyethylene particle-induced osteolysis response in a mouse calvarial model.	International journal of clinical and experimental pathology	Research
Li Cheng, Shengyuan Zhou, Yin Zhao, Yanqing Sun, Zheng Xu, Bo Yuan, Xiongsheng Chen	2018	Tanshinone IIA attenuates osteoclastogenesis in ovariectomized mice by inactivating NF-κB and Akt signaling pathways.	American journal of translational research	Research
Shaowen Zhu, Wanfu Wei, Zhiwei Liu, Yang Yang, Haobo Jia	2018	Tanshinone-IIA attenuates the deleterious effects of oxidative stress in osteoporosis through the NF-κB signaling pathway.	Molecular medicine reports	Research
Fang-Fang Yang, Yu-Hai Gao, Hui-Rong Xi, Wen-Yuan Li, Hui-Ping Ma, Ke-Ming Chen	2018	Effect of Compound Medicine of Tanshinone 2A and Resveratrol on Peak Bone Mass in Growing Rats	Acta Academiae Medicinae Sinicae	Research
Xin Liu, Yumei Niu, Weili Xie, Daqing Wei, Qing Du	2019	Tanshinone IIA promotes osteogenic differentiation of human periodontal ligament stem cells via ERK1/2-dependent Runx2 induction.	American journal of translational research	Research
Yang Wang, Hongyu Chen, Hanyang Zhang	2019	Tanshinone IIA exerts beneficial effects on fracture healing in vitro and in vivo	Chemico-Biological Interactions	Research
Luyao Wang, Liangxing Cheng, Baixia Zhang, Nan Wang, Feng Wang	2019	Tanshinone prevents alveolar bone loss in ovariectomized osteoporosis rats by up-regulating phosphoglycerate dehydrogenase	Toxicology and Applied Pharmacology	Research
Jingjing Zhang, Zixuan Cai, Min Yang, Lijuan Tong, Yan Zhang	2020	Inhibition of tanshinone IIA on renin activity protected against osteoporosis in diabetic mice	Pharmaceutical Biology	Research
